# Metallic Nanoparticles—Friends or Foes in the Battle against Antibiotic-Resistant Bacteria?

**DOI:** 10.3390/microorganisms9020364

**Published:** 2021-02-12

**Authors:** Francisco Amaro, Álvaro Morón, Silvia Díaz, Ana Martín-González, Juan Carlos Gutiérrez

**Affiliations:** Department of Genetics, Physiology and Microbiology, Faculty of Biology, Complutense University of Madrid, 28040 Madrid, Spain; alvmoron@ucm.es (Á.M.); silviadi@bio.ucm.es (S.D.); anamarti@bio.ucm.es (A.M.-G.); juancar@bio.ucm.es (J.C.G.)

**Keywords:** nanoparticles, heavy metals, resistance, antibiotic resistance, oxidative stress

## Abstract

The rapid spread of antibiotic resistances among bacteria demands novel strategies for infection control, and metallic nanoparticles appear as promising tools because of their unique size and tunable properties that allow their antibacterial effects to be maximized. Furthermore, their diverse mechanisms of action towards multiple cell components have suggested that bacteria could not easily develop resistance against nanoparticles. However, research published over the last decade has proven that bacteria can indeed evolve stable resistance mechanisms upon continuous exposure to metallic nanoparticles. In this review, we summarize the currently known individual and collective strategies employed by bacteria to cope with metallic nanoparticles. Importantly, we also discuss the adverse side effects that bacterial exposure to nanoparticles may have on antibiotic resistance dissemination and that might constitute a challenge for the implementation of nanoparticles as antibacterial agents. Overall, studies discussed in this review point out that careful management of these very promising antimicrobials is necessary to preserve their efficacy for infection control.

## 1. Introduction

Multi-drug-resistant bacteria have become one of the most serious threats to public health worldwide [1]. The abuse and misuse of antibiotics have fostered the emergence and transference of resistance mechanisms among bacteria, compromising the therapeutic potential of antibiotics [2]. Among the different strategies that bacteria have evolved to withstand antimicrobial drugs [3], one is their ability to form the so-called biofilms. Bacteria can attach to a surface and grow as a biofilm community where cells aggregate together and surround themselves with a self-produced extracellular matrix that protects them from antibiotics and adverse environmental conditions. Indeed, biofilm-embedded bacteria are up to 100–1000 times more resistant to antibiotics than free-floating planktonic cells [4]. Biofilms can form on any surface, including indwelling medical devices such as catheters or artificial hips, leading to chronic infections that cannot be eradicated with antibiotics.

The rapid spread of antibiotic resistances and the rising prevalence of biofilm-associated infections demand novel strategies to address this challenge, and research has turned to nanomaterials [5,6]. Among them, metallic nanoparticles (NPs) have attracted a great deal of interest because of their special properties such as high reactivity and multiple targets on microbial cells. With many potential applications, some metal-based NPs are already being applied in numerous medical and consumer products, including medical devices, textiles, and cosmetics [5,7]. Moreover, it has been reported that NPs conjugated with antibiotics show synergistic effects against bacteria [5,8]. Therefore, metallic NPs could offer an effective solution for infection control by incorporating them on the contact surfaces of medical devices, textiles, food packages, and membrane filters employed in water treatment [5,9,10], or by being applied topically to treat skin and wound infections [11].

Because NPs display multiple antibacterial mechanisms, it has been thought that bacteria are unlikely to develop resistance against these nanomaterials. However, recent studies have shown that bacteria can in fact tolerate increasing concentrations of copper and silver NPs [12,13,14,15]. Hence, concerns are rising about whether bacteria may develop resistance towards the widely commercialized NP-based antimicrobial materials. While the unique and promising properties of NPs to target bacteria have been covered previously in excellent reviews [5,6,8,9,11], as a novel aspect we highlighted here studies that demonstrated risks associated with bacterial exposure to metallic NPs that may constitute a challenge for their implementation as antibacterial agents. We first describe the experimentally demonstrated mechanisms through which bacteria can gain resistance towards metallic NPs and how that could be overcome. Next, we discuss the different ways by which NPs may favor dissemination of antibiotic-resistant genes among bacteria. Up-to-date research studies in the field were retrieved from Pubmed, SciFinder, and Web of Science databases. The available data point out that careful management of these very promising antimicrobials and further studies addressing how NPs co-select for metal and antibiotic resistance are required.

## 2. Antibacterial Mechanisms of Metallic Nanoparticles

Metallic NPs consist of either metals or metal oxides. Metals such as silver and copper have been recognized for their antimicrobial effects since ancient times [16]. Furthermore, the unique properties of NPs make them promising candidates with high antibacterial activity. For instance, the large surface-to-volume ratio of NPs increases the contact area with bacteria and allows their functionalization with ligands that favor interactions with target bacteria [17]. Although most NP toxicity is attributed to the released metal ions, the antibacterial effect of a NP is highly dependent on its physicochemical characteristics such as size, surface, and charge [18,19]. Importantly, these features can be engineered to maximize bacteria–NP interactions, biofilm penetration, and NP bactericidal efficacy. NP size is a key factor as it determines whether NPs penetrate into bacterial cells and biofilms, thus increasing their toxicity [20]. Smaller particle size (2–10 nm) generally correlates with greater antibacterial effects among NPs with the same metal composition, because of high surface area contact with bacterial cells [21,22,23,24]. Further, NPs smaller than 10 nm in diameter can pass through porins in bacterial membranes, thus exhibiting higher toxicity than larger NPs [25]. NPs smaller than 350 nm have been shown to penetrate through pores within biofilms [20]. Furthermore, NPs have surface charge-dependent toxicity; thus, the more positively charged the NP surface, the more toxic the NP is [6]. The surface charge of NPs determines their interaction with the bacterial surface and biofilms. Indeed, positively charged NPs generally possess better biofilm penetration [26]. In addition, Metch and coworkers recently demonstrated that NP morphology and surface coating influence toxicity and govern NP’s effects on microbial communities [27]. The interface between bacteria and NPs is characterized by electrostatic and hydrophobic interactions. Hence, positively charged and hydrophobic moieties on the NP surface can enhance antibacterial activity [5]. Finally, contact with bacterial components can be influenced by NP shape. In fact, it has been shown that truncated triangular Ag-NPs display greater bactericidal activity than spherical and rod-shaped Ag-NPs [28]. This difference has been attributed to the number of NP facets interacting with the bacterial components. Triangular NPs have more facets than spherical or rod-shaped NPs, and thus cause more cellular damage [5]. In addition, it has been shown that pointed and sharp NPs can pierce bacterial cell membranes, causing cytosolic leakage [28].

**Figure 1:**
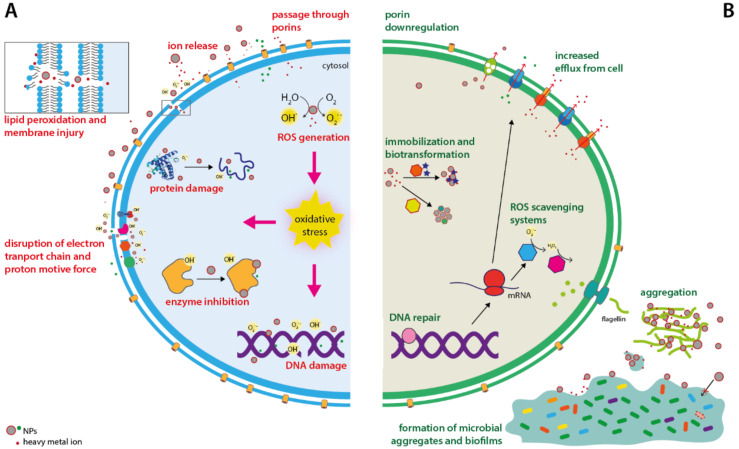
Antimicrobial activities of metallic nanoparticles (**A**) and mechanisms of resistance described in bacteria (**B**). Nanoparticles can damage cell lipids, proteins, and DNA by direct binding or by inducing oxidative stress. To cope with metallic nanoparticles bacteria can reduce metal entry through porin downregulation, overexpressing metal efflux systems, and upregulate antioxidant defenses and DNA repair systems. Furthermore, some bacterial species have evolved mechanisms to modify and immobilize nanoparticles in aggregates with lower toxicity by using enzymatic systems or by secreting polymeric materials that trap nanoparticles in the extracellular environment.

Metallic NPs have proved antimicrobial activity against different microorganisms, including a broad spectrum of both Gram-negative and Gram-positive bacteria [8,17,29,30,31,32,33,34,35,36,37,38]. Despite the large set of experimental data on NP toxic effect, their mechanisms of action are still under debate [39]. Globally, the antibacterial action of NPs is attributed to several causes: (i) cell membrane damage and disruption of membrane potential, (ii) generation of reactive oxygen species (ROS) such as hydroxyl radicals (OH^-^) and superoxides (O_2_^.-^), and (iii) damage to DNA and proteins and inhibition of enzymatic activities [5] (Figure 1A). The negatively charged bacterial cell wall attracts positively charged NPs due to electrostatic interactions. After adhesion onto the bacterial surface, NPs release ions that enter the cell or bind to and destabilize the cell membrane, affecting its permeability and transport activity. ROS generated by NPs oxidize lipids contributing to membrane damage as well [39]. This allows ions and NPs to reach the cytoplasm, where they can induce ROS production, leading to oxidative stress [29,40,41,42,43]. Moreover, ions released from Ag-NPs and Cu-NPs can directly interact with DNA and iron-cysteine clusters present in proteins disrupting their structure and activity [41,44]. Thus, eventually, the extensive damage to a wide spectrum of cell components causes bacterial death.

## 3. Bacterial Resistance Mechanisms towards Metallic Nanoparticles

Because NPs do not need to penetrate into the cell to exert their bactericidal effects, it was proposed that they might overcome antibiotic resistance mechanisms [41]. Furthermore, the non-specific mode of action of NPs toward multiple cellular components suggested that development of resistance is less likely to occur, as bacteria would have to acquire multiple mutations [41,43,45]. In line with this hypothesis, Valentin and coworkers recently showed that *Staphylococcus aureus* more readily developed resistance to ciprofloxacin than to 2-nm Ag-NPs [14]. However, over the last years, a growing body of research has evidenced that bacteria can indeed evolve defense strategies to cope with metallic NPs (Figure 1B). Bacteria with increased resistance to Ag^+^ ions and Ag-NPs have been repeatedly isolated from clinical and non-clinical environments [46,47,48]. More importantly, different researchers have recently proven that resistance to Ag-NPs or Cu-NPs can be quickly induced in vitro in both laboratory and clinical strains after repeated exposure to sublethal doses of NPs. These studies included the ubiquitous *Shewanella oneidensis* and several *Bacillus* species, as well as opportunistic pathogens such as *Escherichia coli*, *Pseudomonas aeruginosa*, or *S. aureus* [13,15,49,50,51,52]. Of particular concern is the fact that rapid Ag-NP resistance development has also been observed in antibiotic-resistant strains [50,53]. Lastly, the ability of bacteria to withstand increasing concentrations of complex metallic NPs (e.g., nickel manganese cobalt oxide NPs) has also been demonstrated in conditions that mimic chronic environmental exposure [49]. Therefore, it is becoming apparent that continuous exposure to sublethal/subinhibitory doses of NPs can favor bacterial resistance. Given the great potential of metallic NPs for infection control, it is urgent to understand how resistance arises; the molecular basis behind it, and to what extent resistance is stable or can be overcome.

The studies published over the last years have shown that bacteria use both individual and collective defense strategies to protect themselves from NPs. While individual cells use their genetically encoded defense mechanisms to withstand NP toxicity, bacteria may also benefit from the additional protection conferred by collective responses, such as the formation of microbial aggregates and biofilms, or the production of substances that immobilize or modify NPs [13,54,55].

Individual strategies mainly involve decreased NP uptake or adsorption, enhanced efflux [56], and detoxification of NP-generated ROS [51,57] (Figure 1B). Cation-selective porins allow for the passage of smaller NPs and NP-released ions through the outer membrane in Gram-negative bacteria [58]. Thus, porin downregulation restricts the access routes of metal ions and renders bacteria less susceptible to NP action. Indeed, loss of porins and overexpression of efflux systems are frequently observed adaptations in Ag-resistant clinical strains isolated from burn wards [46]. Accordingly, different studies have reported downregulation of genes encoding porins in *E. coli* and *P. aeruginosa* exposed to Ag-NPs and Cu-NPs, respectively [12,59]. More importantly, mutations that result in OmpC or OmpF porin deficiency have been associated with increased resistance to Ag-NPs in *E. coli* compared to wild-type strains [60].

In addition to restricting entrance, bacteria use a wide variety of efflux systems to extrude metal ions outside the cell [12,51]. Upregulation of genes encoding efflux pumps in response to NP exposure has been observed in clinically relevant bacteria. For instance, transcriptomic studies carried out by different laboratories proved that *P. aeruginosa* PAO1 overexpressed genes encoding resistance-nodulation-cell division (RND) pumps and other metal efflux systems when exposed to sublethal concentrations of CuO NPs [12] and cadmium quantum dots (Cd-QDs) [56]. Similarly, Ag-NPs were shown to induce the expression of genes encoding copper (*copA*) and copper/silver efflux systems (*cusA*) in *E. coli* as well [8,61]. Importantly, in addition to metal ions, RND efflux systems can expel antibiotics, and thus may confer resistance to both NPs and antibiotics. As it will be discussed later, the widespread use of Ag-NPs or Cu-NPs is raising concerns about potential co-selection of metal and antibiotic resistance [27,62,63].

Furthermore, besides restricting the concentration of metal ions in the cytosol, a number of authors have proposed that bacteria could resist NPs by upregulating their antioxidant mechanisms as well [51]. Indeed, overexpression of genes encoding ROS scavenging systems have been demonstrated in *E. coli, B. subtilis,* and *P. aeruginosa* PAO1 exposed to Ag-NPs, Al_2_O_3-_NPs, and Cd-QDs, respectively [56,57,64], and have been correlated with a better capacity to tolerate prolonged exposure to Ag-NPs [52]. Furthermore, recent work carried out by Valentin and collaborators [14] reported that Ag-NP resistance evolved in *S. aureus* was associated with mutations in genes involved in nucleotide synthesis and oxidative stress defenses.

While individual cells use their genetically encoded defense mechanisms to withstand NP toxicity, bacteria may also benefit from the additional protection conferred by collective responses as stated above. In addition to single or multispecies biofilms, bacteria can also form microbial aggregates with other microorganisms that allow them to withstand NP toxicity and other environmental stresses [54,65]. These aggregates consist of a consortia of microorganisms (such as protists, archaea, or fungi) that are embedded within a self-produced extracellular polymeric substance (EPS) composed of polysaccharides, DNA, or peptides. Biofilms and microbial aggregates offer protection towards NPs in multiple ways. The EPS that forms the aggregate matrix acts as a physical barrier that hinders NP penetration and traps them at the periphery, thus reducing bacteria exposure [20,66], or even modifying the properties of NPs, diminishing their reactivity and their antimicrobial effect [54,66] (Figure 1B). Furthermore, limiting the penetration of NPs may allow bacteria in the interior of the aggregate to sense sub-lethal concentrations and develop an adaptive response that indeed increases NP resistance and enhances biofilm growth [67]. This phenomenon is called hormesis, and it is defined as a process in which exposure to a low dose of a chemical that is deleterious at high doses induces an adaptive beneficial effect on the cell [68]. Hormesis has been observed in different bacteria exposed to some antibiotics and metallic NPs. For example, sub-lethal doses of ZnO-NPs and Ag-NPs were found to promote growth of *P. putida* and *P. aeruginosa* biofilms, respectively, by inducing the expression of quorum sensing and LPS biosynthesis genes as well as the release of signal molecules by bacteria [67,69]. Similarly, *S. oneidensis* MR-1 was reported to increase the production of EPS when exposed to Cu-doped TiO_2_-NPs [70].

## 4. Arming the Enemy: Metallic NPs May Act as Pressure That Co-Selects for Antibiotic Resistance Genes

It has been established that heavy metal exposure enhances the tolerance of bacteria to antibiotics [71,72,73], and that antibiotic-resistant bacteria are abundant in metal-polluted sites [74,75]. Importantly, field studies have described that long-term metal contamination co-selects for antibiotic-resistant genes (ARGs) and metal-resistant genes in different environments such as wastewater treatment plants [21,76], farms [77], and agriculture soils [78,79]. Furthermore, experimental evidence gathered over the last few years suggests that metallic NPs might facilitate the spread of antibiotic resistance between bacteria through co-selection and horizontal gene transference (HGT) of ARGs as well [62,80,81,82]. Hence, given the increasing use of NPs, concern is increasing as prolonged exposure of bacteria to sublethal levels of NPs could act as selective pressure that may accelerate the spread of antibiotic resistance in the environment.

Efforts are now directed at understanding the molecular basis by which NPs could promote the spread of antibiotic resistance. On one hand, metallic NPs could indirectly select for ARGs due to a cross-resistance phenomenon, in which a mechanism initially evolved to tolerate NPs would confer resistance to some antibiotics as well. The upregulation of efflux pumps that can extrude metal ions and antibiotics represents the most conspicuous example of this [71]. The increased abundance of efflux pump genes in soil and water environments under NP exposure has been proven by several studies [14,15,35,56,61,62]. As stated above, some efflux pumps of the RND family are known to export antibiotics in *P. aeruginosa* [83]. On the other hand, metallic NPs could promote horizontal transference of ARGs between bacteria. Over the last years, several studies have found that exposure to sub-inhibitory levels of metallic NPs favors bacterial conjugation and transformation in laboratory cultures [80,82,84,85], natural environments [62,86], and human-made systems [87]. Different laboratories recently observed higher transformation frequency when bacteria were exposed to either ZnO-NP [86] or Al_2_O_3-_NPs [88]. Furthermore, a pioneer study [80] reported that low concentrations of Al_2_O_3_-NPs (nanoalumina) (up to 5 mM) promoted conjugative transfer of the multi-resistance plasmid RP4 between *E. coli* and *Salmonella enteritidis*. Conjugation frequency increased by a factor of 200-fold under Al_2_O_3_-NP exposure compared to the unexposed control cultures. Remarkably, the authors did not find any effect on conjugation frequency when bacteria were exposed to the same concentration of bulk alumina, suggesting a NP-specific effect [80]. Likewise, several studies have described enhanced conjugation transfer of ARGs in bacteria exposed to subinhibitory concentrations of Al_2_O_3_-NPs, ZnO-NPs, TiO_2_-NPs, and CuO-NPs, whereas bulk or ionic CuO, Al_2_O_3_, and TiO_2_ did not [80,84,85,86,89].

Enhancement of HGT by NPs has also been described in “natural environments” as well. Recently, Qi and coworkers reported that rare earth oxide NPs (La_2_O_3_, Nd_2_O_3_, and Gd_2_O_3_ NPs) enriched the abundance of ARGs in NP-amended soils, and that this was accompanied by enhanced tetracycline resistance in the microbial community [82]. The enhanced antibiotic resistance was detected 7 days after soil exposure to NPs and persisted over the whole course of the experiment (60 days). By applying high-capacity quantitative PCR the authors detected a significant increase in the abundance of 24% of the identified ARGs in NP-amended soils when compared to control soils. ARGs were differentially enriched under NP exposure and targeted all major classes of antibiotics [82]. Interestingly, these authors reported a positive correlation between enriched ARGs and the abundance of mobile genetic elements in the NP-amended soils, supporting the hypothesis that NPs might promote the ARG spread through HGT. Although not fully understood yet, the underlying mechanisms appear to involve membrane damage caused by NP-generated ROS and changes in the expression of genes regulating conjugation and transformation. It has been proposed that ROS generated by NPs would lead to both membrane and DNA damage. Indeed, membrane damage and an increased number of conjugative junctions have been observed by transmission electron microscopy (TEM) in *E. coli* and *P. putida* cells exposed to AgO-NPs [81], Al_2_O_3_-NPs [80], or CuO-NPs [89]. On one hand, membrane damage could favor DNA uptake by impairing the membrane barrier. This has been evidenced by different experimental approaches such as flow cytometry, RNAseq, and proteome analysis of conjugating bacteria exposed to CuO-NPs [89] or Ag-NPs [81]. On the other hand, damaged DNA would trigger the SOS response, which in turn might promote HGT by different mechanisms [90]. First, it has been recently proposed that *RecA* overexpression during SOS response might favor recombination of single-stranded DNA with the recipient cell’s DNA [91]. Additionally, RNAseq analysis of NP-exposed cells revealed upregulation of genes encoding the conjugative machinery (such as *trbBp*, *trfAp*) and the SOS response, as well as suppression of genes that repress RP4 conjugation (*korA*, *korB*, and *trbA*) [80]. However, how this occurs has not been elucidated yet. Different studies have shown that sublethal concentrations of ROS-generating antibiotics and biocides promote conjugation via the SOS-response [81,92,93]. Accordingly, the conjugative transfer was found to decrease in the presence of ROS scavengers, supporting the role of ROS in this process [81,89]. Moreover, ROS generation in mating bacteria has been observed with ROS-specific fluorescent dyes, RNAseq, and proteome analysis. For instance, the transcriptomic profile of *E. coli* K12 MG1655 exposed to ZnO-NPs evidenced overexpression of oxidative stress-related genes (*soxS, soxR, oxyR, ahpC*) and SOS response genes (*recX, sbmC, ssb, ada*) [94]. Similar results were described in *E. coli* and *P. putida* exposed to CuO NPs [89].

Lastly, it has been recently reported that NP exposure may contribute to antibiotic resistance by inducing mutations conferring antibiotic resistance. For instance, whole-genome sequencing carried out by Zhang and coworkers [94] found that nanoalumina and ZnO-NPs induced mutations in *gyrA* and *soxR* genes that were associated with resistance to ciprofloxacin and chloramphenicol in *E. coli*. Remarkably, the resistant mutant strains displayed stable resistance to multiple antibiotics. The authors proposed that ROS generated by those NPs led to oxidative DNA damage that in turn triggered the SOS response and error-prone DNA polymerase, resulting in increased mutation rates.

## 5. Strategies to Overcome Bacterial Resistance towards Metallic Nanoparticles

Although a growing body of research has shown that bacteria can readily evolve resistance towards metallic NPs [13,15,95], the genetic basis of NP resistance remains poorly understood. Recent studies reported that NP resistance might evolve through few mutations or without any significant genetic changes in different bacterial species. Graves and collaborators detected few genetic changes in AgNP-resistant isolates of *E. coli* K-12 obtained in laboratory evolution experiments, although the contribution of these mutations to NP resistance has not been investigated yet [15]. In addition, Dong and coworkers reported that mutations in AgNP-resistant populations were mainly associated with the cell surface and two component systems [95]. Recently, Panacek and collaborators showed that three strains of *E. coli* and *P. aeruginosa* evolved Ag-NP resistance by overproducing flagellin, the main flagella protein, to facilitate NP aggregation and immobilization outside cells [13]. Remarkably, while this resistance mechanism was stable over many generations, the authors did not find any mutations in coding sequences in the genomes of the AgNP-resistant isolates. Importantly, the study led by Panacek suggests that the lack of genetic basis of some NP resistance mechanisms could be exploited to counteract bacterial resistance. Indeed, in their pioneer study, the authors were able to overcome Ag-NP resistance in *E. coli* by adding inhibitors of flagellin production such as pomegranate rind extract, [13], paving the way for the use of silver nanoparticles as effective antibacterial agents. Nevertheless, whereas metallic NPs do clearly exhibit tunable features that make them promising antibacterial agents against biofilms and antibiotic-resistant bacteria, further investigation is needed. Caution in the use of NPs for antibacterial purposes is highly recommended, as research published over the last years has proven that continuous exposure to metallic NPs could lead to the development of resistance mechanisms and might contribute to the spread of antibiotic resistances as well.

## Data Availability

Not applicable.
